# Once More the Wrong Method

**Published:** 1914-01-24

**Authors:** 


### THE PROFESSION OF MEDICINE.

### Once More the Wrong Method.

Only last week adverse comment was offered in The Hospital (p. 410) on the manner in which an institutional expert had taken the lay press into his confidence about the subject of radium therapy for cancer. We criticised his actions, partly because his views are unduly optimistic, partly because they were made public in a manner which long experience has shown the medical profession to be undesirable and contrary to public interest. The *Lancet*, which is the recognised organ of all that is soundest and sanest in the profession of medicine, expressed editorially on the same day exactly similar views.

The point that new discoveries in medicine and surgery should properly be communicated in the first instance to recognised medical societies or journals alone, not to sensation-hunting reporters of lay newspapers, should require no labouring. It ought to be self-evident to the most guileless scientist, and much more so to any hospital worker who is a man of the world as well. The principle has, however, been once more conspicuously violated since the Middlesex Hospital surgeons snubbed their director of cancer research; and this time the culprit, or culprits, appear to be connected with Guy's Hospital. So far, they are anonymous; but they are evidently conducting an organised press campaign, which leaves quite in the shade the peccadillo of the indiscreet Middlesex pathologist. On January 14 at least seven newspapers—namely, the *Globe*, the *Evening News*, the *Liverpool Courier*, the *Liverpool Post*, the *Manchester Guardian*, the *Dundee Advertiser*, and the *Daily Citizen*—published paragraphs which in arrangement and general phraseology are evidently of common origin, though the exact wording is not identical. The *Courier's* paragraph was headed "By Private Wire, from our Correspondent"; the *Globe* and the *Evening News* attributed their information to the Central News; the other papers were silent as to the source of their paragraphs.

All four paragraphs are so synoptic that they can be summarised together. The bacteria of the large intestine have long been recognised, so "an eminent surgeon" informed a Central News reporter, as the predisposing cause of many diseases. By some process of reasoning, which is not disclosed as yet, it is concluded that tubercular affections of the joints are thus dependent upon "alimentary toxæmia." Accordingly a child in a very advanced state of joint tuberculosis was deprived of all the large intestine, except nine inches, by a surgical operation at Guy's Hospital; and it speedily recovered from its tuberculosis. Further experiments along the same lines have shown that "certain forms of tuberculosis" can be treated so successfully that colectomy is now "accepted at Guy's as the correct treatment for this disease." This opinion and the cases upon which it is founded are, it is stated, shortly to be communicated to the medical profession. Meanwhile, apparently, their dissemination far and wide in the lay press commends itself to the "eminent surgeon" as a surer way of attaining fame and fortune.

We express no opinion whether the treatment suggested for joint tuberculosis is good or bad: the evidence is not yet made public, and until it is no good purpose can be served by discussing the matter. All that concerns us for the moment is this radically wrong and vicious way of announcing new views on technical medical matters. If the surgeon in question, whoever he may be, is really as eminent as he persuaded the reporter that he is, there is all the less excuse for his grossly unprofessional and improper conduct. So far we have not seen in the press any repudiation of these tactics by the staff of Guy's Hospital. For the reputation of that venerable charity, we venture to suggest that a leaf should be taken from the book of the Middlesex Hospital surgeons. And from the medical profession we certainly look for some authoritative expression of disapproval of this imitation of the theatrical *impresario* by men of standing in the institutional and medical worlds. The Royal Colleges or the General Medical Council might well pay attention to the matter.

If the instances already given are not sufficient proof of the urgent need for improved discipline in the medical profession, yet another serious example of this unethical advertising can be given. On January 13 the *Daily Dispatch*, a Manchester paper, published an account of a wonderful new discovery guaranteed to cure consumption, appendicitis, and goodness knows what else besides. The inventors of this fluid are stated to be a Manchester doctor, aged seventy-four, and a London scientist, who have refused huge sums for their secret, "preferring to offer it to the nation as a free gift for the benefit of suffering humanity."

The aged inventor was obliging enough to talk to a reporter about his discovery. He claims to have treated two hundred cases in six months without a single failure. The nature of the cases is not added; but the statement, at any rate, shows that he has a large practice. He went on to inform the interviewer that he has been peering down microscopes since he was eight years of age.

He even told the pressman that his record of cures sounded almost like the tales of a quack; but he disclaimed all quackery, and if he really intends to publish the details of his discovery in the regular way, that accusation will certainly not be laid against him. As matters stand at the moment, however, he has chosen just exactly that irregular and dangerous method of daily press exploitation which lays him most open to the very suspicion he deprecates. The only other possible explanation of this extraordinary story seems to be that the septuagenarian amused himself by "pulling the leg" of some youthful and gullible reporter. If this is the real truth of the matter, he must be chuckling to himself over the complete success of his joke. But if not, the whole incident serves to show the risk which the medical profession is running of losing its reputation by permitting its members without rebuke to adopt these reprehensible ways and means of publicity.

The Failure of the Panel.

The movement in favour of a State medical service is being furthered in the Ministerial press in a very ingenious way. The strategy of the moment a year ago having required that at all costs the panels should be filled, no stone was left unturned to fill them, somehow or anyhow. Many abuses resulted from the Chancellor's policy. After vehemently asserting for months that the panel system was an unqualified success, the Radical papers are now filled with denunciations of the panels and the panellers, and are making them the basis of a demand for a State medical service.

Certainly some of the facts that are now coming to light are eloquent of what panel service entails. A paralytic doctor, so the *Manchester Guardian* declares, managed to get his name on the panel, and derived a large income by employing an assistant. In other words, the Chancellor's policy involved deliberate encouragement of sweated labour, as well as a covert fraud upon the insured. But, of course, the panels had to be filled; that was the first consideration.

